# Assessing Adipogenic Potential of Mesenchymal Stem Cells: A Rapid Three-Dimensional Culture Screening Technique

**DOI:** 10.1155/2013/806525

**Published:** 2013-02-03

**Authors:** Jean F. Welter, Kitsie J. Penick, Luis A. Solchaga

**Affiliations:** ^1^Department of Biology, Skeletal Research Center, Case Western Reserve University, 2080 Adelbert Road, Millis Science Center, Room 112A, 2080 Adelbert Rood, Cleveland, OH 44106-7080, USA; ^2^Orthopedic Research Lab, University of Arizona, 1501 N. Campbell Avenue, Arizona Health Science Center, Room 8354, Tucson, AZ 85739, USA; ^3^Case Comprehensive Cancer Center, Department of General Medical Sciences and Division of Hematology and Oncology, Case Western Reserve University, Cleveland, OH, USA; ^4^Research and Development, BioMimetic Therapeutics, Inc., 389 Nichol Mill Lane, Franklin, TN 37067, USA

## Abstract

Bone-marrow-derived mesenchymal stem cells (MSCs) have the potential to differentiate into a number of phenotypes, including adipocytes. Adipogenic differentiation has traditionally been performed in monolayer culture, and, while the expression of a fat-cell phenotype can be achieved, this culture method is labor and material intensive and results in only small numbers of fragile adherent cells, which are not very useful for further applications. Aggregate culture is a cell-culture technique in which cells are induced to form three-dimensional aggregates; this method has previously been used successfully, among others, to induce and study chondrogenic differentiation of MSCs. We have previously published an adaptation of the chondrogenic aggregate culture method to a 96-well plate format. Based on the success of this method, we have used the same format for the preparation of three-dimensional adipogenic cultures. The MSCs differentiate rapidly, the aggregates can be handled and processed for histologic and biochemical assays with ease, and the format offers significant savings in supplies and labor. As a differentiation assay, this method can distinguish between degrees of senescence and appears suitable for testing medium or drug formulations in a high-volume, high-throughput fashion.

## 1. Introduction

Much of the research on adult mesenchymal stem cells (MSCs) has been done on bone-marrow-derived populations. First described by Owen and Friedenstein [1], and later more fully characterized by other groups, these cells possess, to some degree, and for a number of population doublings, the defining properties of stem cells, that is, the ability to self-renew and the potential to differentiate along one or more lineages under appropriate culture conditions [1–5]. The chondrogenic, osteogenic, and adipogenic lineages are well documented, but there are likely others [6–10]. The emerging and potentially useful properties of MSCs include their paracrine effects, which may augment the repair of damaged tissues, and their immunosuppressive abilities [11, 12]. With respect to the adipogenic lineage specifically, Mackay et al. have shown that human MSC-(hMSC-) derived adipocytes, express mRNA encoding for adipogenic transcription factors (PPAR*γ*2, C/EBP*α*, and SREBP1), adipokines (adipsin, leptin, APM1, and angiotensinogen), and lipid-metabolizing agents (aP2 and LPL) by day 12 of differentiation and are thus highly analogous to subcutaneous adipocytes at this time point [13]. 

For most of the clinical applications envisioned, a very large number of MSCs will be required [14, 15]. Furthermore, large-scale commercialization of MSCs, where cells from single donors are expanded into thousands of individual doses for use in clinical applications, is emerging. Unfortunately, MSC numbers and differentiation potential, decrease with donor age, and their stem-cell properties are rapidly lost in *in vitro* culture; for example, MSCs senesce and cease proliferating in culture after a limited number of population doublings [16–18]. Even before terminal senescence, the various differentiation potentials are progressively lost [19–21]. Given that these cell properties drift over time, screening populations of MSCs for “stemness” could be an important quality control (QC) consideration, and simple high-throughput assays would be important tools for this screening [22].

Aggregate culture is a cell-culture technique in which cells are induced to self-assemble into three-dimensional tissue-like structures. It is analogous to micromass cultures and has been used successfully to induce and study chondrogenic differentiation of MSCs [23]. We recently adapted the chondrogenic aggregate culture method to a high-throughput 96-well plate format [24–29]. In this paper, we document that adipogenic differentiation can also be achieved in aggregate culture, in the same high-throughput microplate format. Thus, two differentiation potentials can now be verified simultaneously as a part of a potential stem cell QC protocol. Although differentiation of the MSCs still takes a few weeks, the labor and material savings due to the microplate format are considerable. An additional advantage is that aggregates are much less fragile than adipogenic monolayer cultures. They can be picked up and manipulated individually, which simplifies, for example, histologic processing and other assays. Longer-term, this simple, reproducible *in vitro* differentiation model can also be useful for drug screening or toxicology applications.

## 2. Materials and Methods

### 2.1. Materials

Cell culture-media and additives, specifically Dulbecco's Modified Eagle's Medium with 1 g/L glucose (DMEM-LG) or with 4.5 g/L glucose (DMEM-HG), were from Invitrogen (Carlsbad, CA, USA). Fetal bovine serum was from a lot selected as described previously (Sigma Chemical Corporation, St. Louis, MO, USA). Other serum was obtained from Hyclone (Logan, UT, USA). Percoll, dexamethasone, indomethacin, insulin and methyl-isobutyl xanthine (IBMX) were all from Sigma, while ITS^+^ Premix (6.25 *μ*g/mL insulin, 6.25 *μ*g/mL transferrin, 6.25 ng/mL selenious acid, 1.25 mg/mL serum albumin, and 5.35 *μ*g/mL linoleic acid) was from BD Biosciences (Franklin Lakes, NJ, USA). Ascorbate-2-phosphate was from WAKO (Richmond, VA, USA). Other cell-culture additives were from Invitrogen. Polypropylene 96-well microplates and lids were from Phenix (Hayward, CA, USA), while all other cell-culture plasticware was from Becton-Dickinson (Franklin Lakes, NJ, USA). Human recombinant transforming growth factor beta-1 (TGF*β*-1) was from Peprotech (Rocky Hill, NJ, USA), while human recombinant basic fibroblast growth factor (FGF-basic) was donated by the Biological Resources Branch of the National Cancer Institute. Other reagents, unless specifically noted, were from Sigma.

### 2.2. Cells and Cell Culture

#### 2.2.1. Culture Media

MSC expansion medium was 10% FBS in DMEM-LG, either supplemented or not supplemented with 10 ng/mL FGF-2 [9, 30] (note that as in our previous studies, FGF-2 is used only in the expansion medium, not in any of the differentiation media) [30]. The adipogenic induction medium was DMEM-HG with 10% FBS, 1 *μ*M dexamethasone, 100 *μ*M indomethacin, 0.5 mM methyl isobutyl-xanthine (IBMX), 1.745 *μ*M insulin, and the additional supplements listed below. Adipogenic maintenance medium was DMEM-HG with 10% FBS, and 1.745 *μ*M insulin, and the additional supplements. The chondrogenic medium was a defined medium consisting of DMEM-HG supplemented with 1% ITS^+^ Premix, 37.5 *μ*g/mL ascorbate-2-phosphate, 10^−7^ M dexamethasone, and 10 ng/mL TGF*β*-1. Additional supplements such as L-glutamine, antibiotic antimycotic (10,000 units/mL penicillin G sodium, 10 mg/mL streptomycin sulfate, and 25 *μ*g/mL amphotericin B in 0.85% saline), nonessential amino acids, and sodium pyruvate were added to all media at 1%. 

#### 2.2.2. Isolation and Expansion

Human mesenchymal stem cells (hMSCs) were derived from bone-marrow aspirates obtained from 11 healthy volunteer donors at the Hematopoietic Stem Cell Core Facility of the Comprehensive Cancer Center at Case Western Reserve University. Informed consent was obtained, and an institutional review board-approved aspiration procedure was used. hMSCs were isolated as described by Haynesworth et al. [6]. Briefly, the bone-marrow samples were washed with DMEM-LG supplemented with 10% FBS from a selected lot [9]. The marrow sample was centrifuged at 500 ×g on a preformed Percoll density gradient (1.073 g/mL) to isolate the mononucleated cells. These cells were seeded at a density of 1.8 × 10^5^ cells/cm^2^ in a serum-supplemented medium in 10 cm diameter plates. Nonadherent cells were removed after four days by changing the medium. At this point, and for the remainder of the expansion phase, the medium was additionally supplemented with 10 ng/mL rhFGF-2, as described previously [30]. This medium was replaced twice per week thereafter. The primary cultures were subcultured after approximately two weeks and reseeded at 5 × 10^3^ cells/cm^2^ in T-175 flasks. The cells were then used at the end of the first passage. To model replicative ageing and senescence, in some cases, the cells were serially passaged and then used at the end of the third or the ninth passage. In other cases, the cells were expanded to the end of the ninth passage without FGF supplementation. All cell cultures were done at 37°C in a humidified atmosphere of 95% air and 5% CO_2_. Not all preparation were used for all experiments.

#### 2.2.3. Differentiation

hMSCs were induced to differentiate into adipocytes using an adaptation of the media conditions described by Pittenger et al. [3], as modified by Mackay et al. [13]. The culture-expanded hMSCs were used at the end of the first passage, at approximately 80% confluence to prevent contact inhibition and spontaneous differentiation [31]. The MSCs were harvested by trypsinization as described previously: after a rinse with sterile Tyrode's salt solution, 0.25% Trypsin EDTA was added and the cultures returned to the incubator for 5 to 10 minutes [28]. Trypsin was then blocked using bovine calf serum, and the detached cells were centrifuged for 5 minutes at 300 ×g. The supernatant was discarded and the cells were resuspended in one of four medium formulations (I) chondrogenic medium, (II) chondrogenic medium without TGF-*β*1, (III) expansion medium, or (IV) adipogenic induction medium for the first few sets of experiments. For all subsequent work, only adipocyte induction medium was used. 

The cells were counted using a hemacytometer and the suspension volume adjusted to a final cell density of 1.25 × 10^6^ cells/mL. The cell suspension was mixed gently by pipetting, and then 200 *μ*L aliquots (2.5 × 10^5^ cells) were dispensed into the wells of an autoclave-sterilized 96-well, V Bottom, 300 *μ*L polypropylene microplate using a repeater pipette (Eppendorf) with a large orifice tip (Fisher Scientific) to allow smooth delivery of the aliquots into the wells. These were the same plates that we had previously identified as optimal for our microplate chondrogenesis assay [28]. The plate is centrifuged for 5 minutes at 500 ×g and incubated at 37°C in a humidified atmosphere of 95% air and 5% CO_2_. 

Twenty-four hours after seeding, any adherent aggregates were released from the bottom of the wells by aspirating and releasing 100 *μ*L of medium back into the wells using an 8-channel pipette. The medium was changed to adipogenic induction medium on day 2 and every 2-3 days thereafter. From day 12 on adipocyte maintenance medium was used. Given the small volume of medium in each well and the number of cells in each aggregate, adherence to the medium change regimen is important.

### 2.3. Assays

#### 2.3.1. Histology

For the initial aggregation medium formulation experiments, six aggregates from each group were retrieved and formalin-fixed at 1, 2, and 3 weeks after the induction of differentiation. The fixed aggregates were then sequentially infiltrated with 15 and 30% solutions of sucrose in water for 48 hours, embedded in OCT and then snap-frozen in liquid N_2_. Seven *μ*m thick frozen sections were then prepared using a Leica CM1850 cryomicrotome (Leica Microsystems, Bannockburn, IL, USA). The sections were then stained for 8 minutes in Oil-Red O [32]. The Oil-Red O working solution was prepared fresh by diluting a saturated stock solution in isopropanol to 60% with water before each use [32]. The stain solution was 0.2 *μ*m filtered immediately prior to use. Mayer's haematoxylin was used as a counterstain. Sections were then mounted in glycerin jelly and documented at 40x using a Leica DM LB2 upright microscope fitted with a SPOT-RT digital camera [33]. Individual images were then combined into a mosaic of the whole section as described previously [34].

#### 2.3.2. Morphometry

At least ten random 40x digital images were analyzed for each aggregate in each treatment group and time point using the ImageJ software package [35]. Briefly, the total area of aggregate covered in the frame was outlined and measured. The image was then color-thresholded to segment the Oil-Red-stained components [36]. The resulting image was then converted first to 8-bit monochrome and then made binary. Particles were counted with a 50-pixel cutoff to reduce noise. The data are presented as percent area of the aggregate sections stained by Oil-Red. 

#### 2.3.3. DNA Assays

Cell numbers were determined indirectly by measuring the DNA content of the aggregates. Six aggregates were digested individually with papain as described previously [37]. The digested extract was combined with 0.1 N NaOH, incubated at room temperature for 20 minutes, and then neutralized with 0.1 N HCl in 5 M NaCl and 100 mM NaH_2_PO_4_. One hundred microliter of the neutralized mixture was combined with 100 *μ*L of 0.7 *μ*g/mL Hoechst 33258 dye in water. Fluorescence was read using a Tecan Genios Pro plate reader (*λ*_ex_ = 360 nm, *λ*_em_ = 465 nm; Tecan US, Durham NC, USA) and compared to that of a certified calf thymus DNA standard (Amersham, Piscataway, NJ, USA).

#### 2.3.4. GPDH Assays

Glycerol-3-phosphate dehydrogenase (GPDH; EC 1.1.1.8) activity was measured on some aggregates that were harvested at days 0, 1, 7, 14, 21, and 28, using the TaKaRa kit (Clontech, MK426, Mountain View, CA, USA), following the manufacturer's instructions. GPDH catalyzes the reversible reaction between dihydroxyacetone phosphate and glycerol 3-phosphate with NAD as coenzyme; its activity increases during the differentiation of progenitor cells into adipocytes [38]. Briefly, 6 replicate aggregates were washed in PBS and lysed in 500 *μ*L of the lysis buffer solution provided with the kit. A small pestle was used to help break up the aggregate as sonication did not appear to work well. Lysates were stored frozen at −20°C until processed [38]. The lysates were serially diluted with the bis-mercaptoethanol containing dilution buffer. 25 *μ*L of the diluted samples were mixed with 100 *μ*L of substrate at 30°C. The absorbance at 340 nm was then measured every minute for 15 minutes using a Tecan Genios Pro plate reader (Tecan Männedorf, Switzerland) and the supplied UV-transparent 96-well plate. ΔOD_340_ per minute was obtained from dilutions in which the change in OD proceeded linearly, using Sigmaplot software (Systat, San Jose, CA, USA). Activity was computed as prescribed in the kit protocol. Six replicate aggregates from the same donor were analyzed for DNA content, and these values were used for normalization. Confidence intervals for the ratio were estimated using Fieller's theorem.

## 3. Results and Discussion

### 3.1. Results

#### 3.1.1. Aggregate Formation

Regardless of the initial culture medium used (see Section 2.2.3), hMSCs in all four treatment groups and from all donors formed aggregates within the first 24 hours. Similarly, the timing of aggregate formation was identical in P2 and P10 MSCs in these experiments, although this has not always been the case in our hands. However, in P10 cells, this was only the case in MSCs which had been expanded in FGF-2 supplemented medium; those expanded without FGF supplementation failed to form aggregates and thus were not evaluated further. Aside from those cells, there were no gross differences between treatment groups in the aspect of the aggregates. The aggregates were cohesive in that they could be released from the bottoms of the wells by a jet of medium, as described above, without dissociating. Aggregates remained cohesive through 4 weeks although they did compact over time. From the beginning of week 2 and beyond, most aggregates became sufficiently buoyant that they floated to the top of the culture medium in the microplate wells.

#### 3.1.2. Adipogenic Differentiation

**Figure 1:**
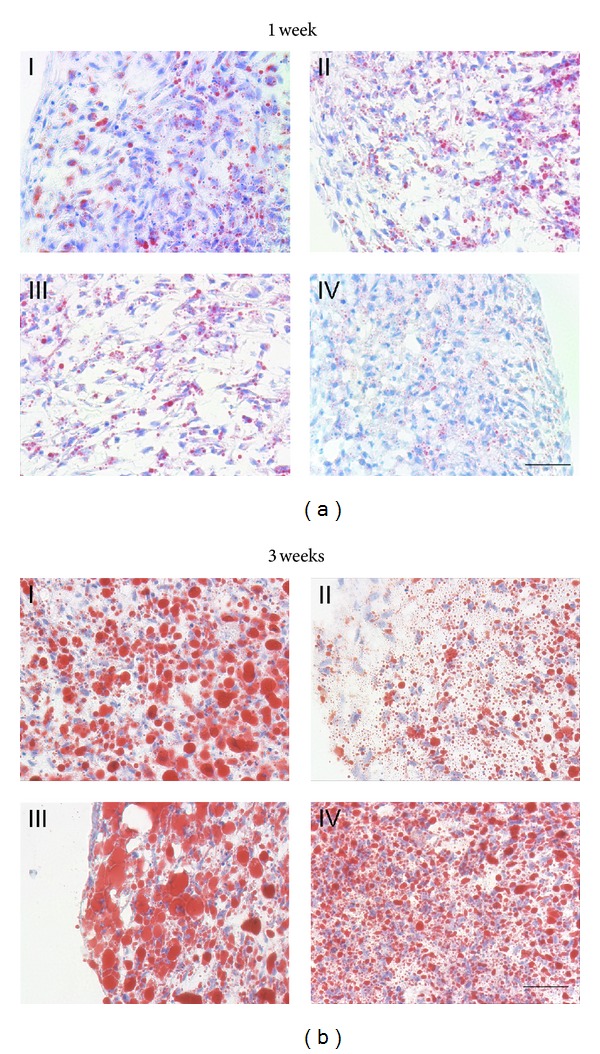
Effect of initial pelleting medium on MSC adipogenesis. Medium compositions were described in Section 2.2.3. (a): At one week (I) 3.3 ± 0.4%, (II) 7.6 ± 0.3%, (III) 6.6 ± 0.8%, and (IV) 5.8 ± 0.8% of the total cross-sectional area were Oil-red O positive (fraction of total aggregate area measured on at least 15 40x fields per condition per donor, *N* = 3). (b): At 3 weeks overall: (I) 50.2 ± 2.3%, (II) 28.5 ± 3%, (III) 58.6 ± 2.4%, and (IV) 47 ± 3.3% of the total cross-sectional area were Oil-red O positive. (fraction of total aggregate area measured on at least 10 40x fields per condition per donor, *N* = 3). Oil-Red O with hematoxylin counterstain, scale bars = 50 *μ*m.

At 1 week, there were differences between the amount of lipid produced by cells initially aggregated in the 4 medium formulations (Figure 1(a)). Compared to adipogenic medium, the differences in aggregate formation in expansion medium or chondrogenic medium without TGF-*β*1 were not significant. By contrast, significantly less (*P* < 0.05) of the aggregate cross-sectional area was Oil-Red O positive in the aggregates formed in complete chondrogenic medium for the first 24 hours (group I). (One-way ANOVA, Dunn's *post hoc* pairwise comparisons).

By three weeks in culture, the fraction of Oil-Red O positive area in group II aggregates was significantly (*P* < 0.01) less than that in the other 3 groups, which were statistically not significantly different from each other. 

**Figure 2:**
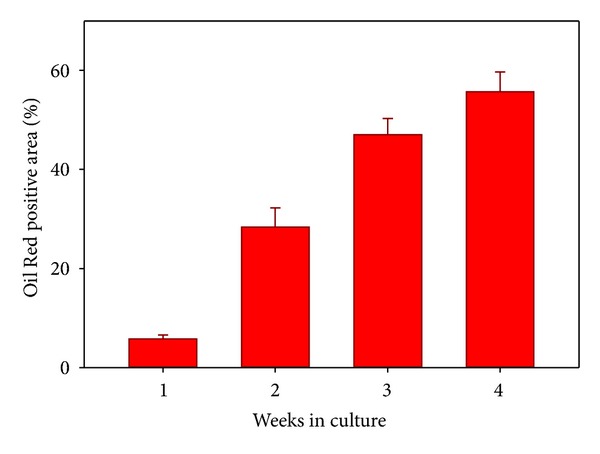
Percent of the cross-sectional area of aggregates which is stained with Oil-Red positive (donors *N* = 3, mean ± SD, and overall 30–60 40x fields were measured for each group). All aggregates were formed in adipogenic medium.

Having established that the cells would form aggregates in adipogenic induction medium, the remainder of the experiments were done using aggregates that were aggregated directly in this medium (Formulation IV). In P2 MSCs, after as little as 1 week of exposure to the adipogenic induction medium, the vast majority of the cells in the aggregates contained several small Oil-Red O-stained lipid droplets (Figures 1 and 2). There was a gradient in droplet size and number, with more numerous and larger drops at the periphery. Overall, about 6% of the cross-sectional area of the aggregates was Oil-Red positive at 1 week.

By two weeks, lipid droplets had increased in size and number. The size gradient from the periphery to the center of the aggregates was more apparent. At this point, about 28% of the aggregate cross section was stained with Oil-Red (Figure 2, histology not shown).

At three weeks, the trend towards larger droplets continued, and the lipid-positive component of the cross-sectional area had increased further to 47%. The trend towards larger and more abundant fat droplets continued at 4 weeks, when 56% of the cross-sectional area of the aggregates was Oil-Red positive (Figures 1(b) and 2).

In all cases, cell numbers, as reflected by DNA content decreased in a time-dependent fashion during the course of the assay (0.95 ± 0.07, 0.71 ± 0.12, 0.45 ± 0.16, and 0.22 ± 0.08 *μ*gDNA, mean ± SD, per aggregate at weeks 1, 2, 3, and 4, resp.).

GPDH activity was below the detection limit of the assay at days 0 and 1. Activity was 11.3 mU/*μ*g DNA by week 1 (90% CI 10.4–12.5), 35.4 mU/*μ*g DNA by week 2 (90% CI 32–39.5), and at week 3 reached 68 mU/*μ*g DNA (90% CI 52.2–87.9).

**Figure 3:**
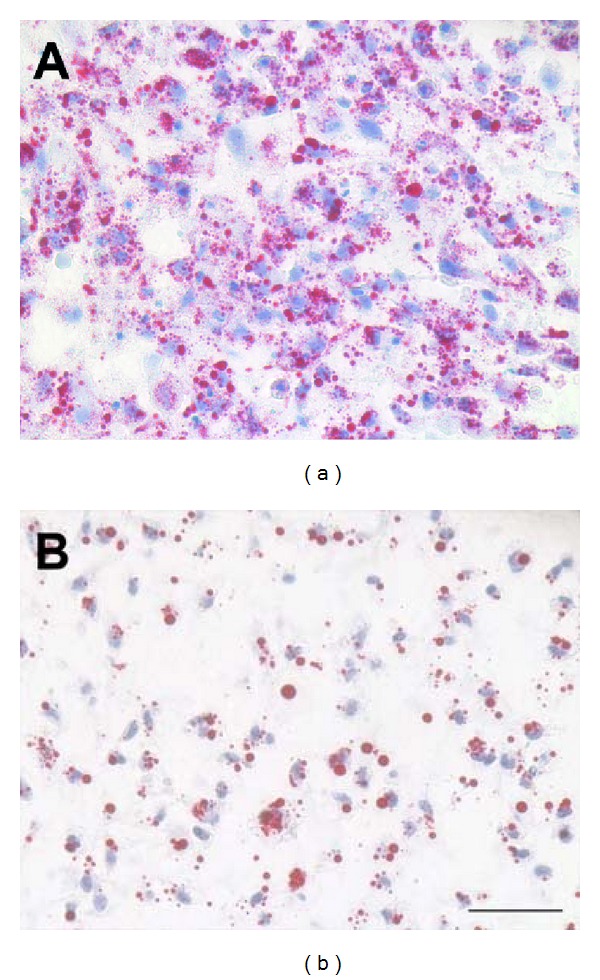
Adipogenic differentiation of hMSCs in aggregate culture at two weeks, illustrating the loss of adipogenic differentiation potential with serial expansion in culture. The cells in both Figures are from the same donor preparation and were expanded using the identical medium formulation. (a): 2nd passage cells; (b): 10th passage cells. On average, passage 10 aggregates had slightly less than half the Oil Red positive area of passage 2 aggregates (*P* < 0.01, 2 donor preparations, 100 40x fields were measured) frozen section, Oil red-O with haematoxylin counterstain. Original magnification: 40x, scale bar: 50 *μ*m.

In MSCs expanded in FGF-2-containing medium and used to form aggregates at P10 (Figure 3), only 50–75% of the cells became Oil-Red positive and both the number and size of the lipid droplets were decreased. Further, the aggregates contracted much less than those from earlier passage cells. hMSCs expanded to P10 in the absence of FGF-2 treatment showed signs of senescence (large surface area, markedly increased doubling time, and prominent stress fibers) and failed to form aggregates at all in adipogenic induction medium.

### 3.2. Discussion

Stem cells are generally defined as having the ability to both self-renew through mitosis and to, under appropriate conditions, differentiate and acquire specialized phenotypes [39]. Stem cell-based tissue engineering and regenerative medicine applications or drug-type cell preparations as used for, for example, graft-versus-host disease, require very large number of cells, which makes extensive and usually rapid *in vitro* subcultivation a requirement. There is, thus, an incentive to maximize the number of cells that can be obtained from a single marrow preparation. Although hMSCs can be expanded to a significant (but nonetheless finite) number of population doublings in culture and can differentiate into a number of mesenchymal phenotypes, both proliferative and differentiation capacities are highly variable [40]. They depend in part on identifiable factors such as the age or health of the donor, the frequency of MSCs in the marrow, the marrow-harvest protocol, and the culture conditions used, but also on yet unexplained donor-to-donor differences [8, 20, 41–44]. In *in vitro *culture, at least, as it is currently practiced, the cells progressively fail in both of the defining “stemness” criteria as they drift and senesce [18, 20, 45, 46]. With respect to proliferation, while there are sporadic reports of 30–40 population doublings (10^9^–10^12^: 1 expansion), preparations grown under comparable conditions frequently cease proliferating after only 4-5 population doublings (50 : 1 expansion) [8, 17, 20, 42]. Well before they lose the ability to proliferate, mesenchymal stem cells also progressively lose the ability to differentiate. The loss of differentiation potentials appears to be a function of passage number, or more importantly, population doubling number [30]. Chondrogenic potential is lost early; the ability to differentiate into adipocytes [20] is more durable, and osteogenic potential is maintained the longest, suggesting this may be the default lineage for bone-marrow-derived MSCs [8, 17, 18, 20, 45, 46]. Given these findings, it does not appear unreasonable to assume that other emerging, and potentially beneficial, properties of MSCs are also lost during extended *in vitro* expansion.

To warrant describing a culture-expanded cell population as mesenchymal stem cells, QC becomes mandatory. Considerations should include documenting the cells' actual differentiation potential at the passage at which they are used in addition to their proliferative potential or cell surface marker expression. Screening techniques based on proxy assays, such as flow cytometry or PCR, may be rapid but may not always be accurate predictors of actual differentiation ability in, for example, tissue engineering applications. Conventional differentiation assays remain cumbersome; we have therefore been adapting MSC differentiation protocols into high-throughput screening assays. 

#### 3.2.1. Screening Assays

Adipogenic differentiation of human MSCs was first described by Pittenger in 1999 [3]. Adipocyte culture is traditionally done in monolayer because of buoyancy even as the so-called ceiling culture or modifications thereof [47, 48]. The latter is a relatively complex and time-, space-, and resource-intensive culture method but works well for MSCs [13]. It results in only small numbers of adherent cells per culture surface area, and these are quite fragile and thus not very useful for further applications. 

Aggregate culture is a simple cell-culture technique in which cells are induced to form three-dimensional tissue structures. It is analogous to micromass cultures and has been used successfully to induce and study chondrogenic differentiation of MSCs [23]. As originally described, individual 15ml conical tubes were used for each aggregate, which was quite cumbersome; we have previously published a high-throughput 96-well plate modification of the chondrogenic aggregate culture method [28, 29]. In this study, we used the same 96-well format as for chondrogenic cultures for the preparation of three-dimensional adipogenic cultures, eliminating the need for fastidious monolayer culture methods. The aggregates are nonadherent and can be handled easily. Histological, biochemical, and enzymatic assays can easily be performed, as described previously.

We had expected that it might be necessary to induce aggregate formation pharmacologically, hence the trials using chondrogenic medium with and without TGF-*β*1 for 24 hours. Somewhat surprisingly, however, the cells formed cohesive aggregates regardless of the initial culture conditions tested. This demonstrates that, on the one hand, three-dimensional aggregation and adipogenic differentiation can be triggered in adipogenic medium directly. On the other hand, for QC purposes, a dual lineage screen can be set up easily as the aggregates in a batch of 96-well plates could be set up from the same cell suspension in chondrogenic medium and then switched to adipogenic medium the next day, thereby eliminating several steps in the setup protocol. Admittedly, there was less lipid deposited in the cells which were initially formed in complete chondrogenic medium, at 1 week (Figure 1(a)), but this difference disappeared by 3 (Figure 1(b)) and 4 weeks (not shown). The cells aggregated in chondrogenic medium, but without TGF-*β*, fared the worst in the long term (Figure 1(b)).

We, and others, have shown previously that human MSCs can be expanded for a far greater number of population doublings in the presence of FGF-2 than without [30]. The effects of FGF-2 supplementation have not only been documented with respect to the retention of proliferative capacity, but also to differentiation capacity [14, 30, 49]. At a basic level, histologic and biochemical analysis can be used in this differentiation assay to easily discriminate between vigorous adipogenic differentiation at P2 and P4, reduced differentiation in FGF-2-treated P10 cells, and nonexistent differentiation in MSCs expanded to P10 without FGF supplementation using histology.

#### 3.2.2. Other Potential Applications

The goal in developing this method was to establish a rapid screening assay for adipogenic potential. Rapid, in the context of this assay, refers to the time commitment required to set up, maintain, and optionally manipulate the cultures. A reasonable number of replicate aggregate cultures, for example, 5–10, can be established by diverting 1-2 × 10^6^ cells. However, a typical MSC preparation can yield 100–250 × 10^6^ cells at the end of passage 1 and 10 times that at the end of passage 2, so it is possible and practical to establish hundreds to thousands of these small-scale adipogenic assays simultaneously from a single marrow aspirate. As, for example, Mackay et al. have shown that human MSC- (hMSC-) derived adipocytes express transcription factors, adipokines, and lipid-metabolizing agents typical of adipose tissue, this approach then has implications for drug, growth factor, and toxicology assays [13]. The medium composition experiments shown here exemplify the type of experiment that can be done easily and in a very compact format. Compared to culture methods like ceiling cultures, the culture medium is readily accessible. Time savings increase with the number of parallel samples for example, for a full 96-well plate, we estimate a 90% reduction in the time needed for common cell-culture tasks such as medium changes compared to cultures done in flasks. Harvesting the cells for assays is done by aspirating the aggregate using a wide-orifice pipet tip. In the future, robotic manipulations and sampling methods can be utilized, further decreasing labor. Although we have not yet explored this possibility, this approach could potentially be developed for use as injectable autologous fat for small-scale applications in cosmetic surgery, for example, not only cosmetic applications in ageing, but also defect-filling after tumor surgery, infections, full-thickness burns, cachexia in AIDS or tumor patients, and so forth. A viable autologous tissue as a filler has clear advantages over the injection of a more-or-less inert foreign substance, but not all patients (burns) actually have fat tissue that is amenable to harvesting. As noted, the aggregates can be handled easily, the large surface to volume area of the individual aggregates mitigates mass-transport issues during culture and provides space for vascular invasion after implantation. Scale-up for larger applications may require a structural scaffold [50]. Combined with MSC cryopreservation, it is possible to envisage multiple implantations over time from a single bone-marrow aspirate, which is not currently possible using autologous native fat.

## 4. Conclusions

In summary, we present a simple method for the establishment and maintenance of large numbers of three-dimensional adipogenic MSC cultures. For general screening of the differentiation potential of MSCs for quality control purposes, both chondrogenic and adipogenic aggregate culture assays can now be done in the same convenient 96-well plate high-throughput format. The method has implications for the refinement of medium formulations, and for adipotropic drug screening, and is sensitive enough to track the loss of adipogenic differentiation potential of cultured MSCs over time. Compared to conventional cell culture, there are significant reductions in labor, space requirements, plasticware, and media costs; further, the use of emerging robotic manipulators would allow for industrial scale-up. To our knowledge, this is also the first practical example of scaffold-free tissue engineering of adipose tissue.
